# A rare case of severe gastroenteritis caused by *Aeromonas hydrophila* after colectomy in a patient with anti-Hu syndrome: a case report

**DOI:** 10.1186/s12879-021-06784-3

**Published:** 2021-10-24

**Authors:** Michael Greiner, Alexia Anagnostopoulos, Daniel Pohl, Reinhard Zbinden, Andrea Zbinden

**Affiliations:** 1 Institute of Medical Microbiology, University of Zurich, Gloriastrasse 28/30, 8006 Zurich, Switzerland; 2 Division of Infectious Diseases and Hospital Epidemiology, University of Zurich, University Hospital of Zurich, Zurich, Switzerland; 3 Division of Gastroenterology, University of Zurich, University Hospital of Zurich, Zurich, Switzerland

**Keywords:** *Aeromononas hydrophila*, Gastroenteritis, Anti-Hu syndrome, Cytotoxic enterotoxin, Case report

## Abstract

**Background:**

*Aeromonas hydrophila* is a gram-negative facultative anaerobic coccobacillus, which is an environmental opportunistic pathogen. *A. hydrophila* are involved in several infectious diseases such as gastroenteritis, septicemia and wound infections. However, gastroenteritis caused by *Aeromonas* spp. are rare and the clinical relevance of *Aeromonas* species in stool specimens is still under debate.

**Case presentation:**

Our case concerns a 32-year-old woman who presented at hospital with a worsening watery diarrhea and fever requiring intensive care. A cholera-like illness was diagnosed. The patient had a past history of an anti-Hu syndrome with a myenteric ganglionitis. A molecular multiplex RT-PCR (QIAstat-Dx Gastrointestinal Panel, QIAGEN) covering a broad spectrum of diverse gastrointestinal pathogens performed directly from the stool was negative but the stool culture revealed growth of *A. hydrophila*. Further investigations of the *A. hydrophila* strain in cell cultures revealed the presence of a cytotoxic enterotoxin.

**Conclusions:**

Although *A. hydrophila* rarely causes gastroenteritis, *Aeromonas* spp. should be considered as a causative agent of severe gastroenteritis with a cholera-like presentation. This case highlights the need to perform culture methods from stool samples when PCR-based methods are negative and gastrointestinal infection is suspected.

## Background

*Aeromonas* spp. can cause different clinical diseases especially in the immunocompromised host. The most common infection sites are wound infections, cellulitis, septicemia and urinary tract infections [1–4]. Gastroenteritis due to *Aeromonas* spp. is generally rare but has been described before in the literature [5–7]. The clinical presentation of gastroenteritis varies from mild diarrhea to shigella-like dysentery to severe cholera-like watery diarrhea [8].

*Aeromonas* spp. are gram-negative facultative anaerobes that are straight, coccobacillary to bacillary cells with rounded ends. Aeromonads usually are oxidase positive and display a fermentative metabolism of glucose. The organisms grow at a range of temperatures from 10 to 42 °C [4]. The genus *Aeromonas* currently consists of 36 species, of which *Aeromonas hydrophila* subsp. *hydrophila*, *Aeromonas caviae*, *Aeromonas dhakensis*, *Aeromonas veronii* biovar sobria (formerly *Aeromonas sobria*) and *Aeromonas trota* are clinically most important and have been isolated often from human feces [9].

Aeromonads feature several virulence factors such as the cytotoxic enterotoxin (Act protein), which has hemolytic, cytotoxic, and enterotoxic activities; type 3 secretion systems and motility factors [10–13]. Enterotoxins play a relevant role in the pathogenesis of diarrhea and their effect is reproducible in animal models [14]. The cytotoxic enterotoxin Act was previously isolated and extensively characterized [15]. Recently, there were also two cytotonic enterotoxins described, a heat stable (Ast protein) and a heat labile enterotoxin (Alt protein) [16, 17]. The presence of Ast and Alt cytotonic enterotoxins in *Aeromonas* spp. were associated with severe diarrhea in children, however, these toxins were found also in environmental strains [16]. To our knowledge, the enterotoxins are chromosomally encoded [10, 11, 18]; the role of plasmids are unknown with the exception of one reported case with evidence of a Shiga-like toxin 1 on a plasmid in strains of *A. hydrophila* [10–13].

The role of *Aeromonas* spp. as enteropathogen is still controversial [19]. We report on a case where severe watery diarrhea was caused by *A. hydrophila* resulting in intensive care medical occupancy. We investigated phenotypically whether the clinical strain isolated from feces produced a cytotoxic enterotoxin.

## Case presentation

A 32-year-old female patient was admitted to hospital with somnolence, aggravation of her chronic diarrhea and fever. The patient had a complicated medical history of intestinal neuronopathy with recurrent pseudo-obstructions due to myenteric ganglionitis. The patient had therefore undergone a hemicolectomy and permanent jejuno-rectostomy. She then developed chronic diarrhea (3 to 4 times per day), which led to a chronic hyponatremia (125 mmol/l, normal value between 135–145 mmol/l). Less than a month prior to hospital admission, anti-Hu antibodies were detected associated with paraneoplastic neurological syndromes [20, 21]. An anti-Hu syndrome with sensory neuronopathy was diagnosed. Despite extensive diagnostics, no underlying malignancies could be found. Immunosuppressive therapy had not yet been initiated.

One week before the current presentation at the hospital, she developed fever, chills, abdominal pain and an increased stool frequency (up to 10 times a day). The diarrhea was of watery consistency without blood or mucus. She had no contact to animals and did not consume contaminated food. None of her social contacts had signs of infectious gastroenteritis. At the emergency room, she was febrile (39.5 °C) and abdominal examination revealed increased bowel sounds without tenderness on palpation. She was somnolent without focal neurological deficits. The laboratory studies showed elevated inflammatory markers (157 mg/L C-reactive protein, CRP, normal value < 5 mg/l), and a severe hyponatremia of 107 mmol/l (normal value between 135–145 mmol/l). No pathological findings were seen on the computed tomography scan of the abdomen, especially no abscess or perforation. The results from a lumbar puncture were inconspicuous and ruled out an infection or inflammation.

The severity of the diarrhea as well as inflammatory markers (CRP max 543 mg/L, procalcitonin, 84.88 µg/L, normal value < 0.1 µg/L) increased quickly despite management in the intermediate care unit. The patient now lost up to 12 L stool per day and was admitted to the intensive care unit for further treatment. On gross examination, her stool was brown and watery. Cultures of the blood, urine and stool were collected and an empiric antibiotic treatment was initiated. With a suspected gastrointestinal focus antibiotic treatment consisted of piperacillin-tazobactam i.v. (4.5 g every 8 h) and vancomycin p.o. (250 mg every 6 h). The blood and urine cultures did not detect any bacterial growth. A molecular multiplex real-time RT-PCR test for detection of numerous gastrointestinal pathogens (QIAstat-Dx Gastrointestinal (GI) panel, QIAGEN, Hilden, Germany) performed directly from the stool was negative (Table 1). The *Clostridioides difficile* glutamate dehydrogenase (GDH) antigen in the stool was positive, as tested by VIDAS *C. difficile* GDH assay (bioMerieux, France), but the toxin genes remained negative as tested by real-time PCR (GeneXpert, Xpert *C. difficile* BT assay, Cepheid, USA).

**Table 1:** Pathogen targets of the QIAstat-Dx Gastrointestinal panel (QIAGEN)

Bacterial targets	Viruses	Parasites
*Clostridioides difficile* toxin A/B Enteroaggregative *E. coli* (EAEC) Enteropathogenic *E. coli* (EPEC) Enterotoxigenic *E. coli* (ETEC) Enteroinvasive *E. coli* (EIEC)/*Shigella* Shiga-like toxin-producing *E. coli* (STEC) Shiga toxin-producing *E. coli* (STEC) O157:H7 *Campylobacter* spp. *Plesiomonas shigelloides* *Salmonella* spp. *Vibrio cholera* *Vibrio parahaemolyticus* *Vibrio vulnificus* *Yersinia enterocolitica*	Adenovirus F40/41 Astrovirus Norovirus GI Norovirus GII Rotavirus A Sapovirus (GI, GII, GIV, GV)	*Cryptosporidium* spp. *Cyclospora cayetanensis* *Entamoeba histolytica* *Giardia lamblia*

After 3 days of treatment without any improvement in the patient’s condition, the piperacillin-tazobactam and vancomycin was stopped and meropenem i.v (1 g every 8 h) and metronidazol i.v. (500 mg every 8 h) was started instead.

For bacterial culture, the stool was incubated on MacConkey’s agar (Oxoid, UK), Columbia 5% sheep blood agar (bioMérieux, Marcy l’Etoile, France) and deoxycholate citrate agar (DCA, Oxoid, UK) at 37 ºC. After 24 h, bacterial growth appeared on the plates showing yellow sucrose fermenting colonies on the DCA plate. The colonies were non-lactose fermenting on DCA and MacConkey’s agar. On the sheep blood agar, bacterial colonies showed a remarkable β-hemolysis. The catalase and oxidase tests both were positive.

The bacteria were identified as *A. hydrophila* by the matrix-assisted laser desorption ionization-time of flight mass spectrometry (MALDI-TOF MS, Bruker Daltonik, Bremen, Germany; using the MALDI Biotyper version 7.0). The antimicrobial susceptibility testing was performed by disk diffusion test on Mueller–Hinton agar plates (MH, Becton Dickinson, Franklin Lakes, NJ) and revealed susceptibility to meropenem, cefepime, piperacillin-tazobactam, ceftriaxone, nalidixic acid, trimethoprim-sulfamethoxazole and amikacin (Table 2). The strain was resistant to amoxicillin and amoxicillin-clavulanate (Table 2)  Interpretative criteria according to the Clinical and Laboratory Standards Institute guidelines, M45 3rd edition, were applied.

**Table 2:** Antimicrobial susceptibility testing of the *A. hydrophila* strain

Antimicrobial agent	Disk content (µg)	Zone diameter (mm)	Interpretive categories and zone diameter breakpoints^a^ (mm)			Interpretation
			S	I	R	
Ampicillin	10	6	n.a	n.a	n.a	Resistant
Amoxicillin-clavulanate	20/10	12	n.a	n.a	n.a	Resistant
Piperacillin-tazobactam	100/10	23	≥ 21	18–20	≤ 17	Susceptible
Ceftriaxone	30	37	≥ 23	20–22	≤ 19	Susceptible
Cefepime	30	33	≥ 25	19–24	≤ 18	Susceptible
Meropenem	10	27	≥ 23	20–22	≤ 19	Susceptible
Nalidixic acid	30	31	n.a	n.a	n.a	Susceptible
Trimethoprim-sulfamethoxazole	1.25/23.75	22	≥ 16	11–15	≤ 10	Susceptible
Amikacin	30	21	≥ 17	15–16	≤ 14	Susceptible

*S* susceptible, *I* intermediate, *R* resistant, *n.a.* not available

^a^According to Clinical and Laboratory Standards Institute guidelines, M45 3rd edition

After the identification of *A. hydrophila*, metronidazol was stopped and following a 10-day treatment with meropenem and intravenous fluid substitution, the patient recovered slowly and hospital discharge was possible. During the next visit, four weeks after discharge, the patient presented without fever or abdominal pain and the frequency of stool was again 3 to 4 times per day.

**Fig. 1:**
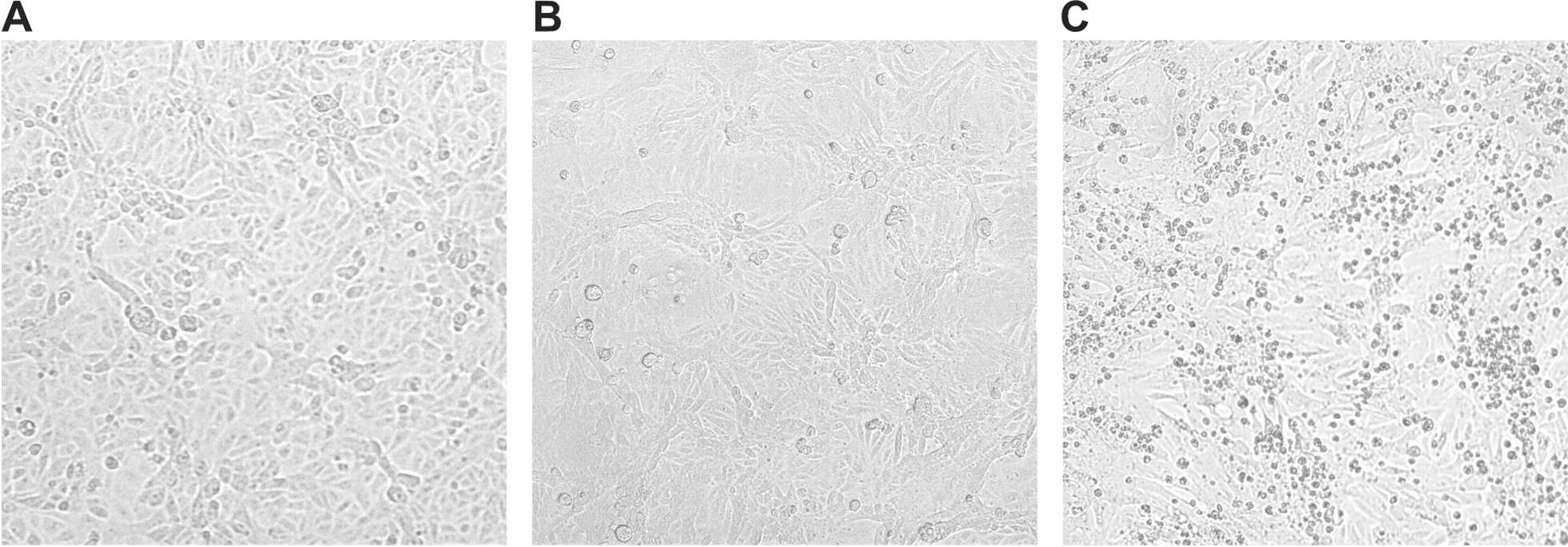
Cytotoxic effects of *A. hydrophila* cultured supernatant in Vero cells **A** negative control with culture media, **B** cytotoxic damage of *A. hydrophila* after 1 day incubation exhibiting cell rounding **C** cytotoxic damage of *A. hydrophila* after 4 days incubation, indicating cellular vacuolation and monolayer destruction

To demonstrate whether the clinical *A. hydrophila* strain produced an enterotoxin, the *A. hydrophila* culture supernatant was analyzed for enterotoxic activity in cell cultures [22, 23]. The bacterial strain was cultivated in trypticase soy broth media (Becton Dickinson, USA). The culture supernatant was sterile filtered and inoculated in a confluent monolayer of Vero (African green monkey kidney) cell lines cultivated in tissue culture tubes with Eagle’s minimum essential medium (MEM, Dulbecco’s, bioswisstec AG, Schaffhausen, Switzerland). Then the tube was incubated at 37 °C in a 5% CO_2_ incubator. Trypticase soy broth and MEM medium were used as negative controls. Cell monolayer morphology was observed using an inverted microscope. After 1 day, morphological alterations in Vero cells were observed inducing rounding, detachment, cellular vacuolation and monolayer destruction (Fig. 1). These observations were consistent with the alterations found in previous reports [22–24]. The negative culture controls did not show these modifications and displayed a confluent monolayer (Fig. 1). The presence of an enterotoxin with cytotoxic activity was suggested.

## Discussion and conclusion

We reported a case of severe gastroenteritis due to *A. hydrophila* in a patient with an anti-Hu syndrome*.* The patient had chronic diarrhea after colectomy, which was exacerbated requiring intensive care. In the literature, severe gastroenteritis caused by *Aeromonas* spp. are described in immunocompromised individuals [25] and in patients with chronic inflammatory bowel disease [26]. In our case, the patient neither received immunosuppressive therapy nor other medications, which would increase the patient’s vulnerability to severe infection. The common differential diagnoses and other pathogens were excluded. To our knowledge, this is the first case of severe gastroenteritis due to *A. hydrophila* in a patient with anti-Hu syndrome.

Despite the association of anti-Hu antibodies with paraneoplastic syndromes, we did not find any underlying tumour in our patient. In a study by Graus et al., a number of patients with neurological paraneoplastic syndromes and anti-Hu antibody positivity in the absence of any tumour were described [27]. In patients such as our patient, with chronic pseudo-obstructions and after ileocoecal surgery, there is a change in the gastrointestinal microbiome [28, 29] and intestinal bacterial overgrowth is more prevalent [30, 31]. Additionally, there is some evidence concerning an increased susceptibility to gastrointestinal infections in patients with a history of gut surgery [32]. Thus, chronic intestinal pseudo-obstructions might be a possible explanation for our patient’s predisposition to severe gastroenteritis with *A. hydrophila*.

Although *Aeromonas* spp. most commonly are isolated in the gastrointestinal tract, their role as an enteropathogen is still controversial [19]. The asymptomatic colonization in developed countries range from 0 to 4% while the isolation rate from stool in persons with diarrheal illness ranges from 0.8 to 7.4% [16, 33]. Nevertheless, there are many case reports that describe *Aeromonas* spp. as a causative enteropathogen [2]. In these reports, either an isolation of the microorganism in the feces or tissue samples was achieved or a positive serological response was present [2].

Beyond any doubt, evidence for local outbreaks is not sufficient, the literature is controversial [34, 35]. In our patient, the source of the infection was not found. *Aeromonas* spp. are environmental opportunistic pathogens that are inhabitants of aquatic ecosystems such as groundwater but might be present also in drinking water or dairy products [36].

In our case, bacterial stool cultures showed growth of *A. hydrophila*. In contrast, a fast multiplex RT-PCR covering a large panel of common gastrointestinal pathogens performed directly from the stool specimen remained negative. In the last years, numerous multiplex systems covering a broad range of gastrointestinal pathogens including bacteria, viruses and parasites appeared on the market. One such system is the QIAstat-Dx GI panel (QIAGEN), which is highlighted by a short turnaround time and was demonstrated to be a valuable tool for diagnosis of gastrointestinal pathogens [37]. Despite the advantages of molecular-based syndromic stool pathogen panels, rare pathogens such as *Aeromonas* spp. are not covered in most syndromic assays including the QIAstat-Dx GI panel [7] (Table 1). Because many laboratories are likely adopting these multiplex syndromic panels and no longer performing stool cultures, gastroenteritis caused by *Aeromonas* sp. might be underestimated.

In our patient, the treatment with piperacillin-tazobactam was not successful in alleviating the symptoms despite proven susceptibility in vitro. A possible explanation might be the presence of chromosomally mediated β-lactamases such as the AmpC β-lactamase, missed by conventional phenotypic tests [38]. In a Korean study analyzing bacteremia caused by *Aeromonas* spp., cases were observed with a piperacillin-tazobactam resistance [39]. When meropenem was installed, clinical improvement and laboratory response were observed. Nevertheless, the use of meropenem is controversial in the treatment of *Aeromonas* spp. due to the possibility of existing chromosomally mediated CphA carbapenemases [38]. Recent reports have identified carbapenemase-producing *Aeromonas* spp. strains in clinical specimens [40].

The production of a cytotoxic enterotoxin is an important virulence factor of *A.°hydrophila* [10, 22, 41]. The presence of the cytotoxic enterotoxin (Act protein) in clinical *A.°hydrophila* strains was previously shown to be associated with cytotoxicity in Vero cells thus indicating the potential of causing severe infections [42]. We have demonstrated the cytotoxic effect of the supernatant of the clinical *A. hydrophila* strain in Vero cells (Fig. 1). Therefore, we speculate that the clinical *A. hydrophila* strain was a cytotoxic enterotoxin producing strain, which was the cause for the severe clinical presentation. A limitation of our case report is, that we have not purified the toxin from the clinical *A. hydrophila* isolate and we have not proved the presence of the *act* gene, which encodes the cytotoxic enterotoxin, by molecular methods.

Although in some gastroenteritis cases the role of the isolation of *Aeromonas* spp. in stool specimens is discussed controversially, in our case, we have proof of the cytotoxic effect of the supernatant of the isolated *A. hydrophila* strain indicative of the presence of an enterotoxin and a remarkable clinical improvement in the patient’s condition after instalment of meropenem. The patient had a decrease in stool frequency, resolution of fever and the inflammatory parameter decreased significantly.

*Aeromonas* spp. should be considered in the differential diagnosis of acute gastroenteritis, which revealed broad spectrum multiplex-PCR negative results. Early diagnosis and initiation of appropriate therapy is crucial for the clinical management. This case might serve as an argument that clinicians should consider also rare causative agents of gastroenteritis and highlights the need to perform culture methods in PCR-negative tested stool specimens where a clinical suspicion of gastrointestinal infection exists.

## Data Availability

All data generated or analysed during this study are included in this published article.

## Abbreviations

CRP: C-reactive protein
MEM: Minimum essential medium
QIAstat-Dx GI panel: QIAstat-Dx Gastrointestinal panel
